# Blood transfusion in deceased donor kidney transplantation

**DOI:** 10.1186/2047-1440-2-4

**Published:** 2013-04-05

**Authors:** Karim Marzouk, Joseph Lawen, Bryce A Kiberd

**Affiliations:** 1 Department of Urology, Dalhousie University, Halifax, Nova Scotia, Canada; 2 Department of Medicine, Dalhousie University, Halifax, Nova Scotia, Canada; 3 Rm 5082 Dickson Building, Queen Elizabeth Health Sciences-VG site, 5280 University Ave, Halifax, Nova Scotia, B3H 1V8, Canada; 4 Rm 294 5th Victoria Building, Queen Elizabeth Health Sciences-VG site, 1276 South Park Street, Halifax, Nova Scotia, B3H 2Y9Canada; 5 Rm 5015 5th Floor Centennial Building, Queen Elizabeth Health Sciences-VG site, 1276 South Park Street, Halifax, Nova Scotia, B3H 2Y9, Canada

**Keywords:** Warfarin, Anticoagulation, Blood shortage, Antiplatelet agents, Transfusion

## Abstract

**Background:**

Given the unpredictable timing of deceased donor organs and the need for blood transfusion, this study was carried out to determine the rate and risk factors for transfusion in order to identifying a low-risk cohort in the face of a critical blood shortage.

**Methods:**

This retrospective chart review examined 306 consecutive deceased solitary kidney transplant recipients from January 2006 to August 2012.

**Results:**

Records show that 80 (26.1%) patients were transfused with a total of 300 units (0.98 units/transplant) during their first hospital stay. Transfusions were higher in patients on warfarin (8/14, 57%, 5.1 units/transplant) and antiplatelet agents (46/136, 33.8%, 1.1 unit/transplant) compared to no anticoagulants (74/156, 16.7%, 0.47 units/transplant). In a multivariable logistic regression analysis warfarin (odd ratio (OR) 8.2, 95% confidence interval (CI) 2.5–27, *P*=0.001), antiplatelet agents (OR 2.9, 95% CI 1.6–5.3, *P*=0.001), recipient age ≥55 years (OR 2.2, 95% CI 1.2–3.9, *P*=0.008), recipient male (OR 0.36, 95% CI 0.2–0.64, *P*=0.001) and preop hemoglobin ≥115 g/L (OR 0.32, 95% CI 0.18–0.57, *P*<0.001) were independent predictors of blood transfusion. Lower bleeding cohorts with transfusion rates <5% could not be identified.

**Conclusion:**

The need for blood is significantly higher in subjects on either warfarin or antiplatelet agents. These patients might be avoided if kidney transplantation is to occur during a critical blood shortage. Unfortunately even patients not on anticoagulation are at some risk.

## Background

The need for blood transfusion after kidney transplantation is not well studied. Rates in one study approached 50% in those receiving deceased donor organs, a rate that seems quite high [1]. The need for blood transfusion with respect to anticoagulation has been addressed in two studies [2,3]. One reported that patients receiving anticoagulation were not at increased risk of bleeding [2], the second concluded the use of warfarin preoperatively was not associated with adverse outcomes in a small case–control series [3]. These observations seem counter to the experience at our center. Lastly the Canadian Committee on Blood and Blood Products issued a framework document on rationing blood during a shortage [4]. The document specifically addressed transplantation and requested that centers collect current rates of blood transfusion in order to categorize high- and low-risk procedures and groups. Knowing risks would aid in individualizing informed consent. The document recommended delaying live donation procedures but allowed deceased donation surgery with informed consent.

The purpose of this study was to examine the prevalence of blood transfusion in deceased organ donor solitary kidney transplantation at our center, to identify risk factors for transfusion need in particular anticoagulation status, and to examine the level of hemoglobin that triggered transfusion. Ideally a low-risk cohort could be identified that could be transplanted in the face of a critical blood shortage to avoid organ waste.

## Methods

Consecutive deceased donor organ transplant recipients of solitary kidneys were identified in the Program’s electronic database from January 2006 until November 2012. Combined organ and living donor transplants procedures were excluded. Data were extracted from our electronic health records which came into existence in January 2006. Approval for this retrospective study was obtained from our institution’s research ethics board.

All patients received induction with either basiliximab or antithymocyte globulin (ATG) and all received preoperative solumedrol (500 mg) intravenously. ATG was reserved for those who were repeat transplant recipients, deceased cardiac donor recipients, and the highly sensitized. Postoperative patients received oral tacrolimus, mycophenolate, and prednisone. All patients were treated with sulfamethoxazole-trimethoprim for pneumocystis prophylaxis and oral rantidine for gastrointestinal ulcer prophylaxis. Patients on a proton pump inhibitor pre transplant were continued on this agent in place of ranitidine. All patients were to receive subcutaneous heparin 5000 units twice or thrice daily for venous thromboembolism prophylaxis. Patients on warfarin received vitamin K and fresh frozen plasma to reduce their INR to <1.5. However two patients received a prothrombin complex to reduce their INR. No therapy was given to patients on antiplatelet agents.

Data extracted included packed red cells transfused, hemoglobin at transfusion, anticoagulation on admission (warfarin, antiplatelet use, or both), erythropoiesis stimulating agents, cause of ESRD, diabetes mellitus, age, gender, delayed graft function (DGF as defined as need for dialysis post transplantation), days in hospital, cold ischemic time, CMV status, HLA sensitization as defined by most recent panel reactive antibody (PRA,%), reversal of anticoagulation therapy (in the case of warfarin treated patients, postoperative prophylaxis). Patient data for transfusions were limited to the first hospitalization or to 30 days whichever came first.

Variables associated with transfusion need were examined by binary logistic regression analysis. Variables statistically associated with transfusion in a univariable analysis were studied in a multivariable model. For age and pre-transplantation hemoglobin receiver-operator characteristic curves were used to identify values with the best sensitivity and specificity. Significance was assumed at 5%. Inappropriate transfusion was defined as initiation of therapy for hemoglobin >90 g/L. Statistical analysis was performed using IBM SPSS Statistics software version 20.0.

## Results

There were 306 patients in the cohort. Blood transfusion occurred in 80 (26.1%) patients for a total of 300 units (0.98 units/transplant). Characteristics associated with the need for blood are shown in Table 1. Transfusion rates (Table 2) were higher in patients on warfarin (8/14, 57%, 5.1 units/transplant) and antiplatelet agents (46/136, 33.8%, 1.14 units/transplant) compared to those not on anticoagulants (26/156, 16.7%, 0.47 units/transplant). Overall transfused patients received 3.8±3.8 units. Slightly more than half (44/80) of patients transfused received either 1 (*n*=14) or 2 (*n*=30) units. Only 18 (5.9%) patients received ≥5 units and six received ≥10 units. Of the 18 patients requiring 5 or more units 15 were either on antiplatelet agents or warfarin. Therefore only three (1.9%) patients on no anticoagulants, nine (6.6%) on antiplatelet agents, and four (29%) on warfarin required had a large transfusion requirement (≥5 units). Of the patients on antiplatelet agents almost all were on aspirin and only four were on clopidigrel.

**Table 1:** Patient characteristics stratified by blood transfusion

	**No transfusion ( *n *=226)**	**Transfusion ( *n *=80)**	**Probability**
Age (years)	50 ± 13	55 ± 12	0.002
Male gender (%)	149 (66)	37 (50)	0.008
Diabetes mellitus (%)	45 (20)	27 (34)	0.012
HLA MM	4.1 ± 1.3	4.2 ± 1.4	0.451
cPRA (%)	14 ± 27	20 ± 32	0.634
Hb pre g/L	121 ± 14	114 ± 13	<0.001
Platelet count	230 ± 73	230 ± 88	0.980
Donor age (years)	47 ± 7	50 ± 16	0.137
Donor sex male (%)	91 (40)	45 (56)	0.014
CIT (h)	12.5 ± 6.3	11.8 ± 5.3	0.403
Antiplatelet (%)	90 (40)	46 (58)	0.006
Warfarin (%)	6 (3)	8 (10)	0.007
Hb nadir g/L	88 ± 13	69 ± 10	<0.001

**Table 2:** Patient cohort by anticoagulation status

	**All patients ( *n *=306)**	**Warfarin ( *n *=14)**	**Antiplatelet agents ( *n *=136)**	**No anticoagulant ( *n *=156)**	**Probability**
Age (years)	51 ± 13	50 ± 10	55 ± 11	48 ± 13	<0.001
Male gender	186 (61)	10 (71)	93 (68)	83 (53)	0.021
Diabetes mellitus (%)	72 (24)	1 (7.1)	56 (41)	15 (9.6)	<0.001
cPRA (%)	16 ± 28	14 ± 29	14 ± 26	18 ± 30	0.484
HLA MM	4.1 ± 1.4	3.4 ± 1.6	4.2 ± 1.2	4.2 ± 1.2	0.082
CIT (h)	13.1 ± 10.8	12.4 ± 6.1	14.2 ± 14.4	11.8 ± 6.2	0.084
Donor age (years)	48 ± 17	52 ± 17	51 ± 15	45 ± 17	0.006
Donor gender male	136 (44)	6 (43)	62 (46)	68 (44)	0.936
Hb pre g/L	119 ± 14	122 ± 14	119 ± 14	119 ± 14	0.658
INR	1.03 ± 0.22	1.89 ± 0.36	0.99 ± 0.10	0.98 ± 0.08	<0.001
Platelet count	230 ± 77	230 ± 94	233 ± 85	227 ± 70	0.828
Transfusion (%)	80 (26)	8 (57)	46 (33.8)	26 (16.7)	<0.001
Units total (units/patient)	300 (0.98)	71 (5.1)	155 (1.14)	74 (0.47)	<0.001
Hb nadir g/L	83 ± 15	77 ± 23	81 ± 14	85 ± 14	0.018
Length of hospital stay (days)	12.5 ± 10.2	18.7 ± 13.7	13.9 ± 12.9	10.6 ± 5.9	0.001

Age ≥55 years and hemoglobin ≥115 g/L were determined to have the best sensitivity and specificity to predict the need for a blood transfusion. In a multivariable logistic regression analysis warfarin (OR 8.2, 95% CI 2.5–27, *P*=0.001), antiplatelet agents (OR 2.9, 95% CI 1.6–5.3, *P*=0.001), recipient age ≥55 (OR 2.2, 95% CI 1.2–3.9, *P*=0.008), recipient male (OR 0.36, 95% CI 0.2–0.64, *P*=0.001) and pre op hemoglobin ≥115 g/L (OR 0.32, 95% CI 0.18–0.57, *P* <0.001) were independent predictors of blood transfusion. Most patients received venous thromboembolism prophylaxis (281 or 92% of the cohort) and this treatment was not associated with bleeding (OR 0.92, 95% CI 0.34–2.4, *P*=0.87). Diabetes mellitus was significant in a univariable analysis but not in the multivariable analysis.

The mean hemoglobin at transfusion was 68±8 g/L. Only 9 units were transfused for a hemoglobin ≥90 g/L and 21 for a hemoglobin level between 80 and 89 g/L. Therefore 90% of transfused units were for a hemoglobin level <80 g/L. Transfusion decisions were not consistent as 60 patients had nadir hemoglobin levels <80 g/L and were not transfused.

Lower bleeding cohorts could be identified however the reduction in blood use was minimal. For example, those on no anticoagulants and male sex reduced the cohort to 83 patients (27%) with a transfusion rate of 10.8% (0.37 units/transplant). Those on no anticoagulants and a hemoglobin ≥115 g/L reduced the cohort to 31% of the overall population sample with a transfusion rate of 12.5% (0.36 units/transplant). Restricting those on no anticoagulants and recipient age <55 years reduced the cohort to 36% of the sample with a transfusion rate of 13.6%. Men, no anticoagulants and hemoglobin >114 g/L represented only 51 patients (16.7% of the cohort) with a transfusion rate of 9.8% (0.26 units/transplant). The lowest transfusion cohort consisted of men <55 years of age, no anticoagulants and hemoglobin ≥115 g/L. These represented only 40 patients (13% of the entire cohort) and 7.5% were transfused.

## Discussion

Based on this analysis patients on anticoagulants especially warfarin are at particularly increased risk of requiring blood transfusion post kidney transplantation. This analysis shows that transfusion is also not uncommon (16.7%) in those not on anticoagulants. Smaller cohorts could be identified but this greatly restricted access without greatly reducing the need for blood.

This analysis was restricted to deceased donors since live donation could be delayed during a blood shortage. Transfusion was also higher in male donors (OR 1.9, *P*=0.014) however this was not included in the analysis since most would want to proceed with the transplantation regardless to avoid organ waste. Donor age was not significantly associated with transfusion. There may be other factors that correlate to transfusion including the presence of heart disease. Unfortunately this was not specifically examined. Those who were on antiplatelet agents were almost certain to have vascular disease or be at high risk for cardiovascular events. Older patients were more likely to be transfused and this might have been due to a perceived higher risk of vascular disease. Patients at high risk of cardiovascular events might also be avoided during a critical blood shortage but this was not specifically examined.

The frequency of blood transfusion was greater than appreciated but in keeping with other studies. In a single center from the US, Scornik *et al*. found rates of transfusion to be 51% of deceased donor kidney recipients and 30% of live donor recipients [1]. In our study 26% of deceased donors and 14% of 209 live donor kidney recipients over the same time period (data not shown) were transfused. In another single center US study, 25% of kidney recipients were transfused. A UK study reported 45% of patients on warfarin and 29% of non-warfarin-treated patients required blood [3]. In aggregate our transfusion rates appear to the same or lower than these reports.

The impact of anticoagulation of transfusion rates was analyzed in two of the above studies. The UK study as noted above reported that those on warfarin were at no increased risk (45% *versus* 29%) [3]. However it is not clear if the controls included subjects on antiplatelet agents and given the small sample size a significant difference may have been missed. In the larger study by Eng *et al*. pre transplant anticoagulation was not associated with the need for transfusion. However patients receiving postoperative heparin required more blood. Their study may have included live and deceased donor recipients [2]. Analyzing both live and deceased donors may have reduced the likelihood of detecting an effect of anticoagulation on transfusion rates in deceased donors. In an analysis of our live donors anticoagulation with antiplatelet agents or warfarin was also not associated with bleeding (data not shown). More of our patients were on antiplatelet agents (136 of 306) compared to the Eng study (69 of 327). In the Eng study those on antiplatelet agents who were transfused tended to receive on average more units (3.5 units for clopidogrel, 3.3 units for ASA, and 2.5 units for no anticoagulation). Differences between centers may well be an important determinant of transfusion practice and rates.

It is not clear how appropriate our transfusion practice approached the ideal. A recent meta-analysis of blood transfusion practice did not find any adverse effects of a restrictive policy of blood transfusion compared to a liberal policy [5]. Unfortunately none of the 19 studies were of kidney transplant recipients but did include a variety of subjects with acute bleeding in the context of surgery. Adverse effects, including 30-day mortality, stroke, myocardial infarction, and length of stay, showed a trend to be less in the restricted arm. For the most part our practice approached the restrictive spectrum (mean hemoglobin at transfusion was 68 g/L) however 10% were transfused at more liberal rates (hemoglobin >90 g/L). A more restrictive policy may have reduced the need of transfusion in our center without compromising outcomes. The effects of blood transfusion on new HLA antibody formation appears to be low in the short term [1]. However the cumulative effect of transfusions may compromise outcomes in those needing a repeat transplant [6].

## Conclusions

Given the above, restricting access to deceased kidney transplantation to those not on anticoagulants might be prudent during a critical blood shortage. Patients undergoing transplantation during a blood shortage should be informed of their risk. At our center the risk is small (2%) for a large bleed but modest (16.7%) for the probability of needing some blood for those not on anticoagulants. The rates are considerably higher for those on antiplatelet agents and warfarin. Each center should review their transfusion practice and know their transfusion rates to better inform their patients.

## Abbreviations

ATG: Antithymocyte globulin; CI: confidence interval; CIT: Cold ischemic time; CMV: Cytomegalovirus; cPRA: Calculated panel reactive antibodies; Hb: Hemoglobin; HLA MM: Human leukocyte antigen mismatch; INR: International normalized ratio; OR: Odds ratio

## Competing interests

The authors declare they have no competing interests.

## Authors’ contributions

KM collected data and helped draft manuscript. JL co-designed study, critically appraised the analysis and significance. BK co-designed study, collected and analyzed data, assisted in manuscript preparation. All authors read and approved the final manuscript.
